# Supplementary material for: Surface darkening by abundant and diverse algae on an Antarctic ice cap
Source: Nat Commun. 2025 Mar 18;16:2647. doi: 10.1038/s41467-025-57725-6 (PMC11920422; doi:10.1038/s41467-025-57725-6)
Supplement: Supplementary file 1 — Supplementary Information [file 41467_2025_57725_MOESM1_ESM.pdf]

Supplementary Figures and Tables

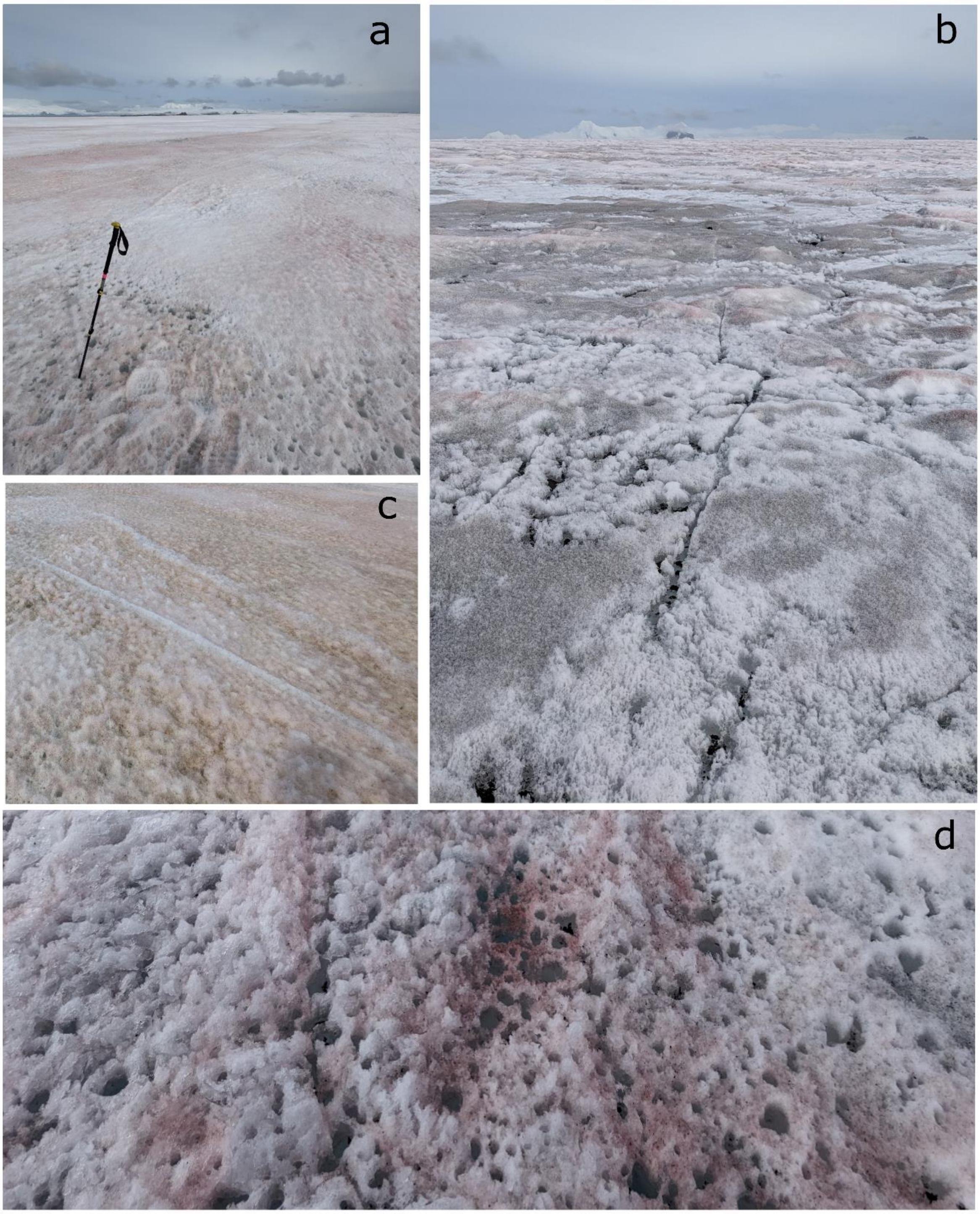

Supplementary figure S1a. Example photographs of the Robert Island ice cap, Antarctica, surface snow and ice*. (Photo credit AG).*

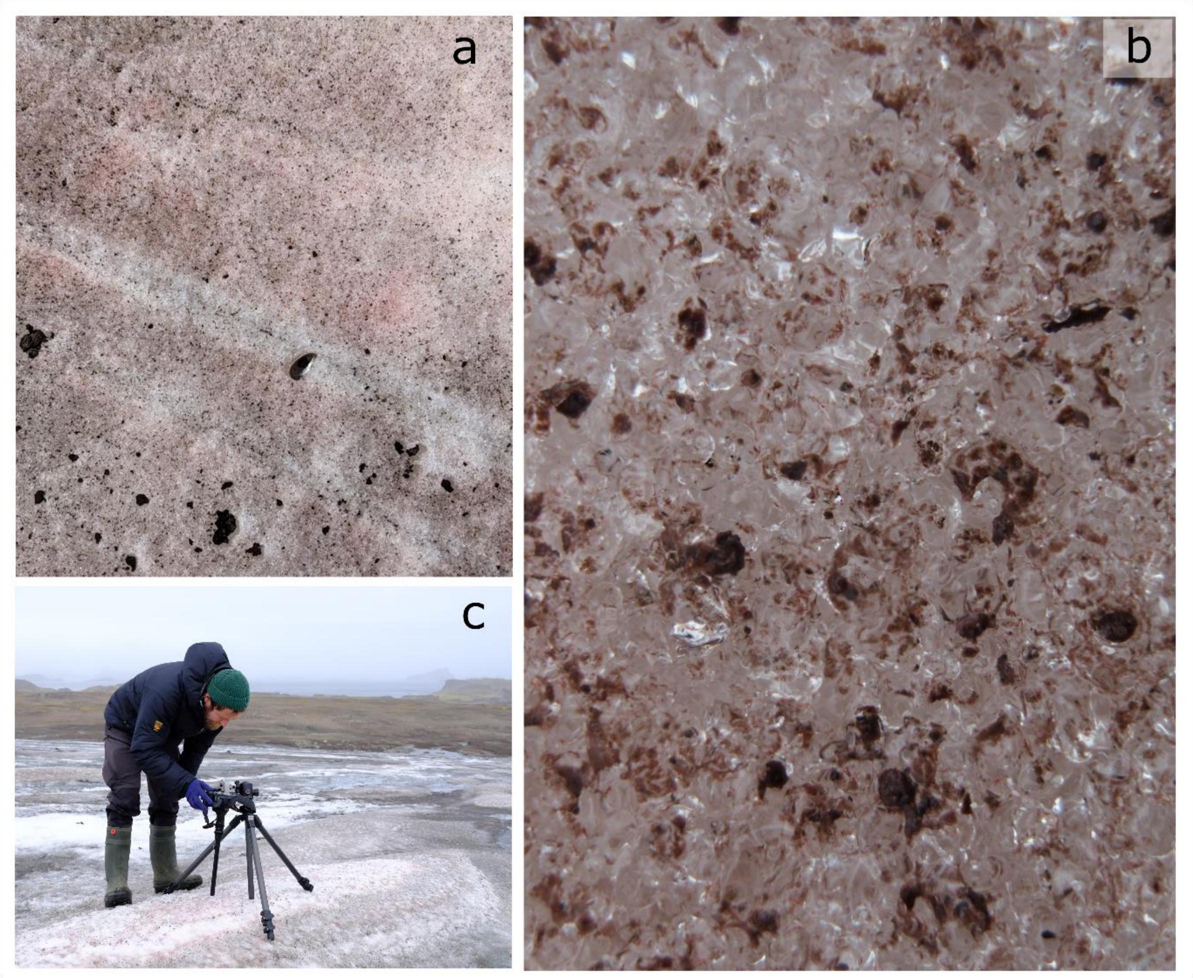

Supplementary figure S1b. Example imagery of Robert Island ice cap surface composition. *a) Ice cap surface, showing typical névé-like snow-ice with strong red and glacier algae pigmentation. b) Macro-photography of snow ice-crystal structure at the same location. Individual cells can be seen attached to the surface of ice crystals. c) Location of Dino-Lite imagery in Fig. 1C, and macro-photography in this figure (a. and b.). (Photo credit AT, AG).*

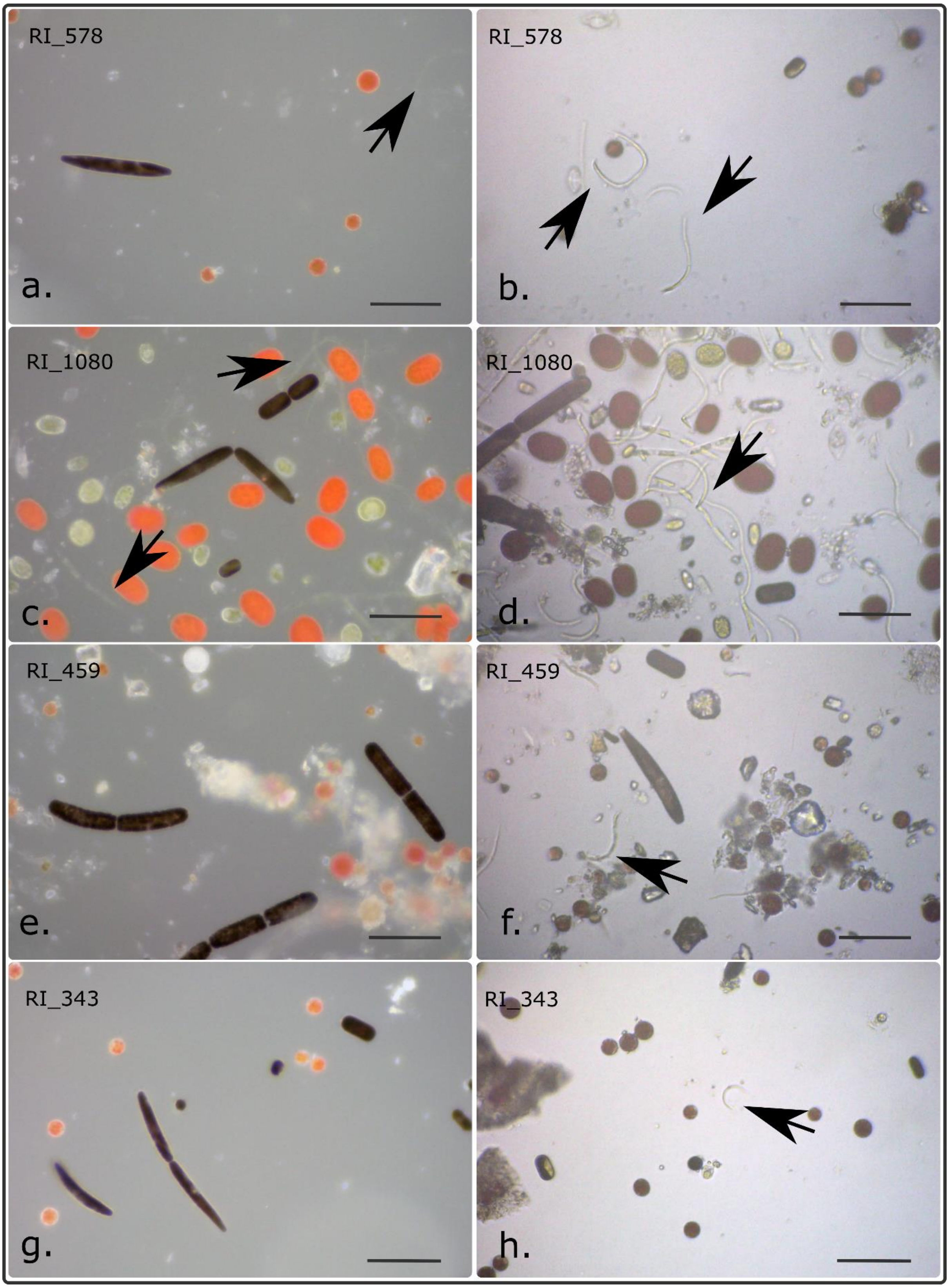

Supplementary figure S2. Representative Dinolite micrograph images of algal communities observed from the Robert Island ice cap (South Shetland Islands, Antarctica). *Micrographs of fresh samples imaged at the field station 1- 5 h after collection. Scale bars 50 µm. Right hand images were taken using the Dinolite autoexposure setting to improve contrast for imaging filamentous* Raphidonema-*like cells. Arrows point to examples of* Raphidonema-*like filamentous cells. Photo credit (AT).*

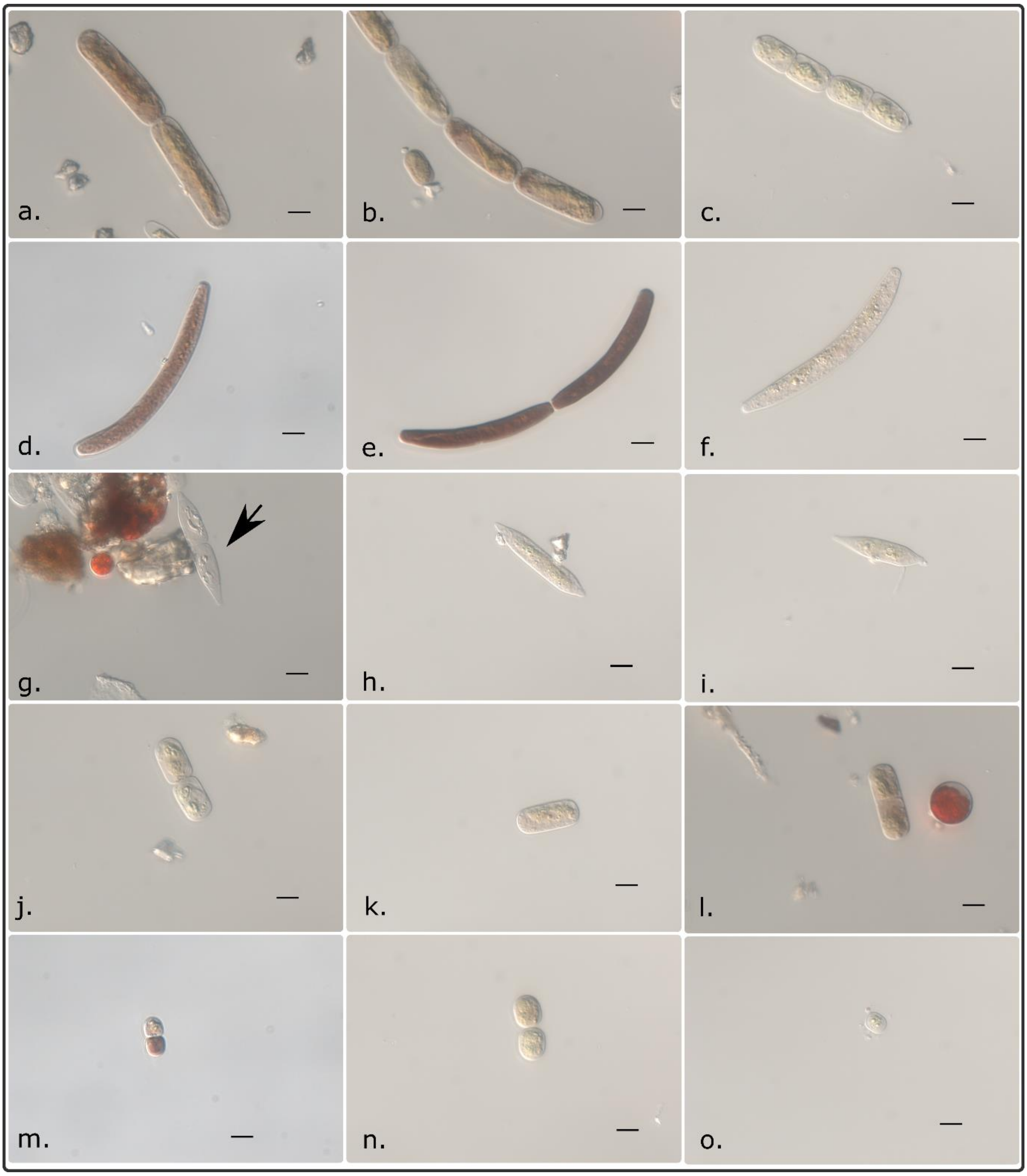

Supplementary figure S3. Brightfield micrograph images of* Ancylonema *morphological diversity from the Robert Island ice cap (South Shetland Islands, Antarctica). *Images taken on an AxioImager 1 with Axiocam 506 colour camera system six months after sampling and on sample delivery to the UK. Scale bars represent 10 µm.* Ancylonema *cells showed noticeable loss of pigmentation despite storage at <-20°C throughout the period of transport. Photo credit (AT).*

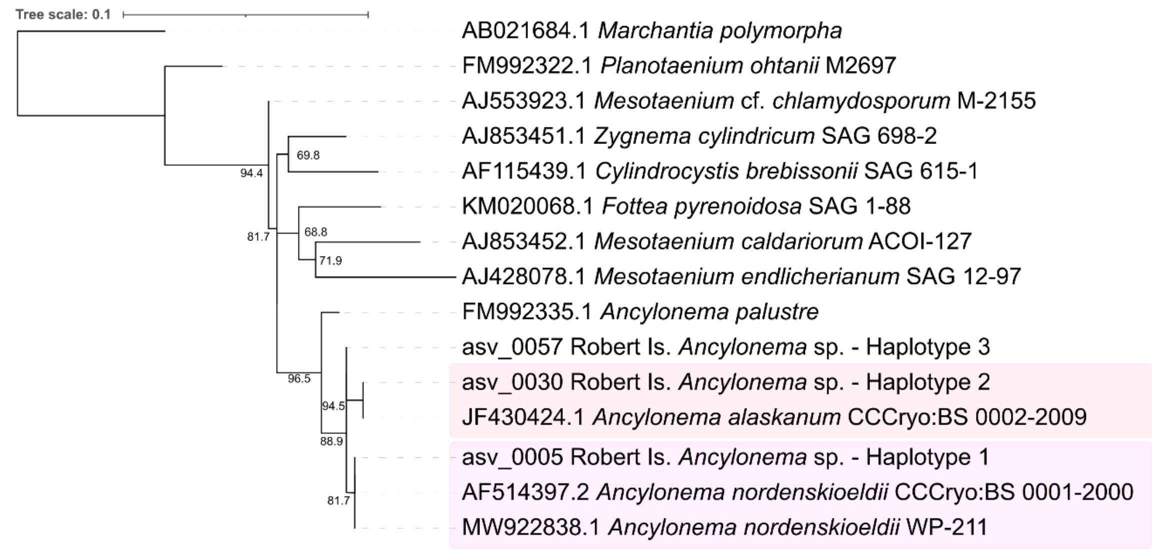

Supplementary figure S4. 18S V4 phylogeny of Robert Island* Ancylonema *haplotypes. *Phylogenetic consensus neighbour-joining tree of 18S V4 sequences (1000 bootstrap) from Robert Island* Ancylonema *haplotypes, as well as reference sequences for* A. nordenskioeldii *and* A. alaskanum*, and a wider selection of Zygnematophyceae reference sequences, adapted from a previous phylogenetic analysis (Procházková et al., 2021)* . Marchantia polymorpha *represents the outgroup. Clades putatively ascertaining to* A. nordenskioeldii *and* A. alaskanum *are indicated in colour.*

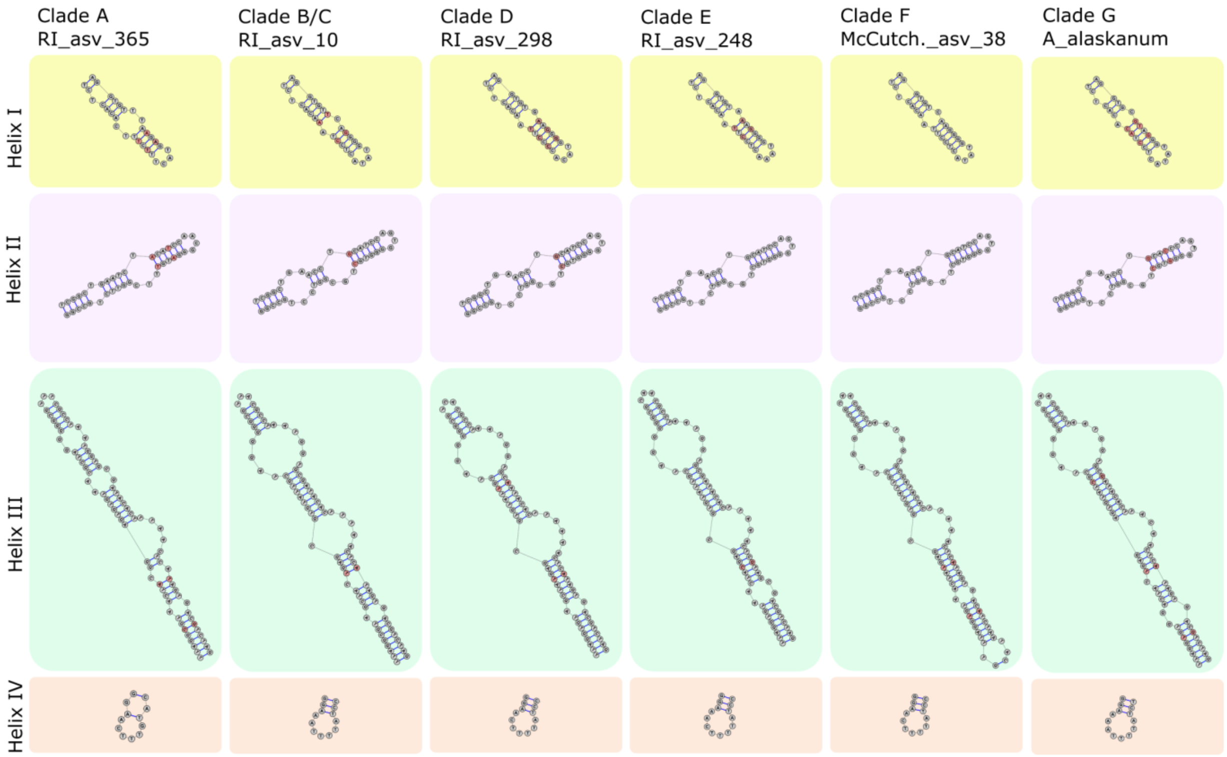

Supplementary figure S5. ITS2 secondary sequence-structure maximum expected accuracy fold predictions. *Schematics showing variation in structure in helices I, II, III, and IV for representative* Ancylonema *from assigned clades. Variant CBC positions are highlighted in red.*

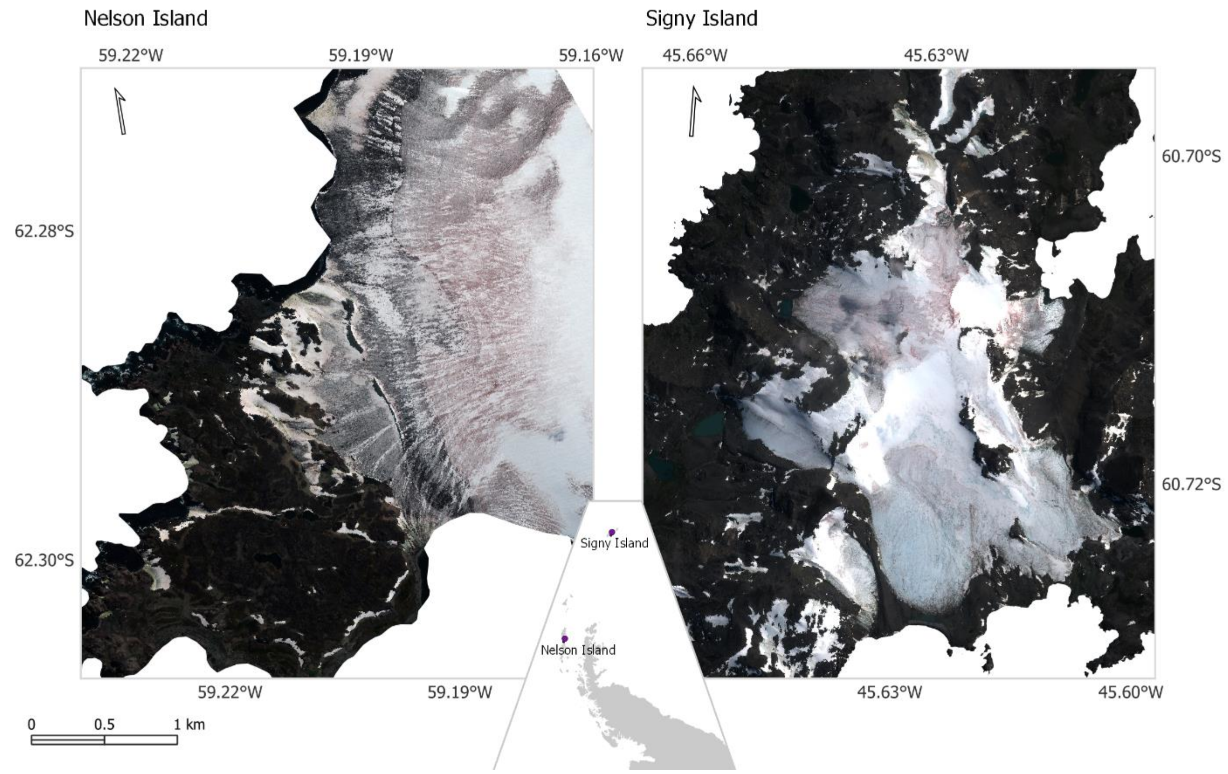

Supplementary figure S6. Example imagery of putative ice cap algal blooms in the Antarctic region. *Worldview imagery of Nelson Island (3 February 2021) and Signy Island (21 January 2021) showing conspicuous ice cap surface blooms.*

Supplementary table S2. 18S and ITS2 haplotypes. *Haplotype ASV numbers, assigned clades, read counts, and proportion of total reads (from respective datasets) for that marker.*

Study	Marker	ASV	Haplo-type	Clade	Reads	Percent-age
Robert Island	18S_V4	asv_00005	1	n/a	271913	9.7
Robert Island	18S_V4	asv_00030	2	n/a	46185	1.6
Robert Island	18S_V4	asv_00057	3	n/a	27152	1.0
Robert Island	ITS2	asv_00010	1	C	130263	5.9
Robert Island	ITS2	asv_00019	2	C	62818	2.9
Robert Island	ITS2	asv_00020	3	E	57531	2.6
Robert Island	ITS2	asv_00043	4	C	34531	1.6
Robert Island	ITS2	asv_00069	5	G	15169	0.7
Robert Island	ITS2	asv_00080	6	C	13609	0.6
Robert Island	ITS2	asv_00088	7	D	12161	0.6
Robert Island	ITS2	asv_00094	8	C	10942	0.5
Robert Island	ITS2	asv_00120	9	C	8243	0.4
Robert Island	ITS2	asv_00136	10	B	7075	0.3
Robert Island	ITS2	asv_00154	11	B	5802	0.3
Robert Island	ITS2	asv_00155	12	C	5659	0.3
Robert Island	ITS2	asv_00194	13	E	3964	0.2
Robert Island	ITS2	asv_00222	14	G	3133	0.1
Robert Island	ITS2	asv_00240	15	C	2750	0.1
Robert Island	ITS2	asv_00248	16	E	2756	0.1
Robert Island	ITS2	asv_00254	17	E	2504	0.1
Robert Island	ITS2	asv_00298	18	D	2124	0.1
Robert Island	ITS2	asv_00315	19	C	1786	0.1
Robert Island	ITS2	asv_00365	20	A	1451	0.1
Segawa et al., 2018	ITS2	asv_00094	n/a	E	2395	0.7
Segawa et al., 2018	ITS2	asv_00179	n/a	D	854	0.3
Segawa et al., 2018	ITS2	asv_00259	n/a	D	357	0.1
McCutcheon et al., 2021	ITS2	asv_00024	n/a	E	13805	0.5
McCutcheon et al., 2021	ITS2	asv_00038	n/a	F	5329	0.2
McCutcheon et al., 2021	ITS2	asv_00042	n/a	F	4752	0.2
McCutcheon et al., 2021	ITS2	asv_00055	n/a	E	3368	0.1

Supplementary table S3. Additional ITS2 environmental sequence datasets. *Location, SRA dataset numbers, and distribution of reads for the additional ITS2 environmental datasets and ASVs analysed.*

**Location**	**SRA dataset(s)**	**Latitude**	**Longitude**	Segawa_asv_094	Segawa_asv_179	Segawa_asv_259	McCutcheon_asv_024	McCutcheon_asv_038	McCutcheon_asv_042	McCutcheon_asv_055
Greenland (Kangerlussaq)	SRX6813812 to SRX6813830	67.22888 N	49.12305 W	0	0	0	13805	5329	4752	3368
Greenland (Qaanaaq)	DRX122002	77.89473 N	68.77335 W	2020	22	0	0	0	0	0
Greenland (Ilulissat)	DRX122003	69.56110 N	49.34715 W	273	698	0	0	0	0	0
Alaska (Gulkana)	DRX122011	63.28068 N	145.41242 W	0	57	0	0	0	0	0
Svalbard (Longyearbyen)	DRX122004	78.13747 N	16.15794 E	67	0	357	0	0	0	0
Svalbard (Ny Alesund)	DRX122008	78.89904 N	11.82895 E	35	77	0	0	0	0	0
			**Total Reads**	2395	854	357	13805	5329	4752	3368

Supplementary table S5. Comparing cell densities from glacier algae blooms worldwide. *A comparison of maximum recorded* Ancylonema *spp. cell counts* ml^{-1} *from glacier algal studies.*

Study	Location	Cells ml^{-1}
Robert Island (this study)	Robert Island ice cap, Antarctica	**2.1×10^5**
Ling and Seppelt, 1990	Windmill Islands, Antarctica	**1.0×10^5**
Procházková et al., 2021	Gurgler Ferner, Austria	**4.8×10^5** ($\pm 0.07 \times 10^5$) (*A. alaskanum*)
Procházková et al., 2021	Morteratsch Glacier, Switzerland	**4.8×10^5** (± 0.25 x 10^5) *(A. nordenskioeldii)*
Millar et al., 2024	Morteratsch Glacier, Switzerland	**9.0×10^5**
Di Mauro et al., 2020	Morteratsch Glacier, Switzerland	**0.24×10^5** (mean)
Yallop et al., 2012	Greenland Sermersuaq	**2.9×10^5**
Cook et al., 2020	Greenland Sermersuaq	**0.29×10^5** ($\pm 0.2 \times 10^5$) (mean of high biomass)
Stibal et al., 2017	Greenland Sermersuaq	**1.8×10^5**
Williamson et al., 2018	Greenland Sermersuaq	**0.16×10^5**
Remias et al., 2012	Longyearbreen, Svalbard	**0.24×10^5** (mean)

Supplementary References

Cook, J. M., Tedstone, A. J., Williamson, C., McCutcheon, J., Hodson, A. J., Dayal, A., ... and Tranter, M., 2020. Glacier algae accelerate melt rates on the south-western Greenland Ice Sheet. *The Cryosphere*, 14(1), 309-330.

Di Mauro, B., Garzonio, R., Baccolo, G., Franzetti, A., Pittino, F., Leoni, B., Remias, D., Colombo, R. and Rossini, M., 2020. Glacier algae foster ice-albedo feedback in the European Alps. *Scientific Reports*, 10(1), p.4739.

Ling, H. U., and Seppelt, R. D., 1990. Snow algae of the Windmill Islands, continental Antarctica. *Mesotaenium berggrenii* (Zygnematales, Chlorophyta) the alga of grey snow. *Antarctic Science*, 2(2), 143-148.

McCutcheon, J., Lutz, S., Williamson, C., Cook, J. M., Tedstone, A. J., Vanderstraeten, A., Wilson, S., Stockdale, A., Bonneville, S., Anesio, A. M. and Yallop, M. L., 2021. Mineral phosphorus drives glacier algal blooms on the Greenland Ice Sheet. *Nature Communications*, 12(1), p.570.

Millar, J. L., Broadwell, E. L., Lewis, M., Bowles, A., Tedstone, A. J. and Williamson, C. J., 2024. Alpine glacier algal bloom during a record melt year. *Frontiers in Microbiology*, 15, p.1356376.

Procházková, L., Řezanka, T., Nedbalová, L., and Remias, D. (2021). Unicellular versus filamentous: The glacial alga *Ancylonema alaskanum* comb. et stat. nov. and its ecophysiological relatedness to *Ancylonema nordenskioeldii* (Zygnematophyceae, Streptophyta). *Microorganisms*, 9(5), 1103.

Remias, D., Holzinger, A., Aigner, S., and Lütz, C., 2012. Ecophysiology and ultrastructure of *Ancylonema nordenskiöldii* (Zygnematales, Streptophyta), causing brown ice on glaciers in Svalbard (high arctic). *Polar Biology*, 35, 899-908.

Segawa, T., Matsuzaki, R., Takeuchi, N., Akiyoshi, A., Navarro, F., Sugiyama, S., Yonezawa, T. and Mori, H., 2018. Bipolar dispersal of red-snow algae. *Nature Communications*, 9(1), p.3094.

Stibal, M., Box, J. E., Cameron, K. A., Langen, P. L., Yallop, M. L., Mottram, R. H., ... Ahlstrøm, A. P.,2017. Algae drive enhanced darkening of bare ice on the Greenland ice sheet. *Geophysical Research Letters*, 44, 11,463–11,471. https://doi.org/10.1002/2017GL075958

Williamson, C. J., Anesio, A. M., Cook, J., Tedstone, A., Poniecka, E., Holland, A., Fagan, D., Tranter, M. and Yallop, M. L., 2018. Ice algal bloom development on the surface of the Greenland Ice Sheet. *FEMS Microbiology Ecology*, 94(3), p.fiy025.

Yallop, M. L., Anesio, A. M., Perkins, R. G., Cook, J., Telling, J., Fagan, D., MacFarlane, J., Stibal, M., Barker, G., Bellas, C. and Hodson, A., 2012. Photophysiology and albedo-changing potential of the ice algal community on the surface of the Greenland ice sheet. *The ISME Journal*, 6(12), pp.2302-2313.
