# Supplementary material for: Surface darkening by abundant and diverse algae on an Antarctic ice cap
Source: Nat Commun. 2025 Mar 18;16:2647. doi: 10.1038/s41467-025-57725-6 (PMC11920422; doi:10.1038/s41467-025-57725-6)
Supplement: Supplementary file 3 — Reporting Summary [file 41467_2025_57725_MOESM3_ESM.pdf]

nature portfolio

Corresponding author(s): Alex Innes Thomson, Andrew Gray, Matthew P. Davey

Last updated by author(s): 2024-08-15

Reporting Summary

Nature Portfolio wishes to improve the reproducibility of the work that we publish. This form provides structure for consistency and transparency in reporting. For further information on Nature Portfolio policies, see our Editorial Policies and the Editorial Policy Checklist.

Statistics

For all statistical analyses, confirm that the following items are present in the figure legend, table legend, main text, or Methods section.

n/a	Confirmed	
☐	☒	The exact sample size (*n*) for each experimental group/condition, given as a discrete number and unit of measurement
☐	☒	A statement on whether measurements were taken from distinct samples or whether the same sample was measured repeatedly
☒	☐	The statistical test(s) used AND whether they are one- or two-sided<br>*Only common tests should be described solely by name; describe more complex techniques in the Methods section.*
☒	☐	A description of all covariates tested
☒	☐	A description of any assumptions or corrections, such as tests of normality and adjustment for multiple comparisons
☐	☒	A full description of the statistical parameters including central tendency (e.g. means) or other basic estimates (e.g. regression coefficient) AND variation (e.g. standard deviation) or associated estimates of uncertainty (e.g. confidence intervals)
☒	☐	For null hypothesis testing, the test statistic (e.g. *F*, *t*, *r*) with confidence intervals, effect sizes, degrees of freedom and *P* value noted<br>*Give P values as exact values whenever suitable.*
☒	☐	For Bayesian analysis, information on the choice of priors and Markov chain Monte Carlo settings
☒	☐	For hierarchical and complex designs, identification of the appropriate level for tests and full reporting of outcomes
☒	☐	Estimates of effect sizes (e.g. Cohen's *d*, Pearson's *r*), indicating how they were calculated

Our web collection on statistics for biologists contains articles on many of the points above.

Software and code

Policy information about availability of computer code

Data collection	All molecular reference sequences and SRA environmental sequence datasets were accessed through the NCBI public database portal:<br>https://www.ncbi.nlm.nih.gov/
Data analysis	Environmental sequence data were processed using the publicly available R package dada2 v1.26.0 in R v 4.2.1 "funny-looking-kid"<br>Phylogenetic analysis was undertaken using the IQTree web-server and IQ-TREE multicore version 1.6.12<br>Instantaneous radiative forcing calculations were performed in R Studio version 2022.02.3<br>Remote sensing analysis was undertaken in QGIS<br>All code can be made available upon request

For manuscripts utilizing custom algorithms or software that are central to the research but not yet described in published literature, software must be made available to editors and reviewers. We strongly encourage code deposition in a community repository (e.g. GitHub). See the Nature Portfolio guidelines for submitting code & software for further information.

Data

Policy information about availability of data

All manuscripts must include a data availability statement. This statement should provide the following information, where applicable:

- Accession codes, unique identifiers, or web links for publicly available datasets
- A description of any restrictions on data availability
- For clinical datasets or third party data, please ensure that the statement adheres to our policy

Field data, including cell count data, cell measurement (biometric) data and remote sensing outputs will be made available through the UK BAS Polar Data Centre upon acceptance. Environmental sequence data will be submitted and made available from the NCBI Sequence Read Archive (SRA) upon acceptance.

Research involving human participants, their data, or biological material

Policy information about studies with human participants or human data. See also policy information about sex, gender (identity/presentation), and sexual orientation and race, ethnicity and racism.

Reporting on sex and gender	*Use the terms sex (biological attribute) and gender (shaped by social and cultural circumstances) carefully in order to avoid confusing both terms. Indicate if findings apply to only one sex or gender; describe whether sex and gender were considered in study design; whether sex and/or gender was determined based on self-reporting or assigned and methods used. Provide in the source data disaggregated sex and gender data, where this information has been collected, and if consent has been obtained for sharing of individual-level data; provide overall numbers in this Reporting Summary. Please state if this information has not been collected. Report sex- and gender-based analyses where performed, justify reasons for lack of sex- and gender-based analysis.*
Reporting on race, ethnicity, or other socially relevant groupings	*Please specify the socially constructed or socially relevant categorization variable(s) used in your manuscript and explain why they were used. Please note that such variables should not be used as proxies for other socially constructed/relevant variables (for example, race or ethnicity should not be used as a proxy for socioeconomic status). Provide clear definitions of the relevant terms used, how they were provided (by the participants/respondents, the researchers, or third parties), and the method(s) used to classify people into the different categories (e.g. self-report, census or administrative data, social media data, etc.) Please provide details about how you controlled for confounding variables in your analyses.*
Population characteristics	*Describe the covariate-relevant population characteristics of the human research participants (e.g. age, genotypic information, past and current diagnosis and treatment categories). If you filled out the behavioural & social sciences study design questions and have nothing to add here, write "See above."*
Recruitment	*Describe how participants were recruited. Outline any potential self-selection bias or other biases that may be present and how these are likely to impact results.*
Ethics oversight	*Identify the organization(s) that approved the study protocol.*

Note that full information on the approval of the study protocol must also be provided in the manuscript.

Field-specific reporting

Please select the one below that is the best fit for your research. If you are not sure, read the appropriate sections before making your selection.

☐ Life sciences ☐ Behavioural & social sciences ☒ Ecological, evolutionary & environmental sciences

For a reference copy of the document with all sections, see nature.com/documents/nr-reporting-summary-flat.pdf

Ecological, evolutionary & environmental sciences study design

All studies must disclose on these points even when the disclosure is negative.

Study description	This study reveals the large-scale presence, diversity, and bioalbedo effects of algae on Antarctic ice caps, highlighting their impact on melting dynamics and their previously unstudied role in maritime Antarctica. Remote sensing data, validated by point sampling of algal bloom density and spectral characteristics, enabled the estimation of bloom extent across the Robert Island ice cap surface, facilitating the calculation of the theoretical contribution to ice melt from algal biological albedo reduction (BAR). Microscopy (n = 698 cells from 37 samples) combined with environmental sequencing (8 pooled samples) characterised the composition of the algal community, and revealed a large and unique diversity of within the genus Ancylonema in Antarctica, indicative of regional-endemic processes within Antarctic cryoflora communities.
Research sample	The research sample consists of point and landscape-scale ice surface data from a specific area of the Robert Island ice cap in the South Shetlands archipelago of maritime Antarctica, focusing on algal blooms occurring on the ice cap surface. Sampling was confined to weathering crust areas of the Robert Island ice cap that were accessible by foot from the research station (Base Luis Risopatron, INACH, Chile). The sampling effort concentrated on regions with high bloom presence, primarily within the ice cap ablation zone, and extended up to the upper snow line, beyond which significant algal blooms were presumed to be absent.
Sampling strategy	Point samples for cell density, spectral data, microscopy, and environmental sequencing were selected ad hoc from the ice cap surface. Samples for spectral and cell count data were collected from various surface types, identified by ice coloration, representing a gradient from low to high algal biomass. Environmental sequencing samples were chosen based on successful DNA amplification, which depended on input biomass and the success of DNA extraction. Due to the dangers of working on an ice-cap a fully randomised sampling strategy could not be undertaken.

Data collection	Field data from Robert Island was collected by co-authors AIT, AG, HM, and MD.
Timing and spatial scale	Field samples were collected during one austral summer season, from the 7 January 2023 to 14 February 2023. Point sampling were conducted ad hoc throughout the period, typically once a week, whenever weather conditions permitted. Cell count and measurement data were gathered from samples collected across this time frame. The sampling area covered 13.7 km² of the ice cap, with point sampling restricted to areas accessible on foot and free from hazards such as supraglacial rivers and crevasses.
Data exclusions	Areas beyond the ice cap, including snow and soil regions, were not included in this study. Samples that failed amplification during the preparation of sequencing libraries were excluded from the sequencing process. Additionally, ASV sequences that fell below the stated confidence thresholds were excluded from the final analysis.
Reproducibility	This study represents an observational dataset collected over a single season, rather than an experimental study. Reproducibility of the findings across different years and at different sites within the region should be a priority for future research. To validate the reproducibility of measurement approaches, technical replicates were included for cell counts, microscopy, and environmental sequencing.
Randomization	Semi-randomised. Samples on the ice cap were chosen in areas of algal presence, with areas where no algae were visible also sampled. Due to safety on the glacier a full grid randomised sample approach was not possible.
Blinding	Blinding was not relevant to this work. The study represents an observational dataset, rather than an experimental study.

Did the study involve field work? ☒ Yes ☐ No

Field work, collection and transport

Field conditions	Fieldwork was conducted in January and February 2023 from the Luis Risopatron field station on Robert Island (INACH, Chile). UAV remote sensing was carried out on suitably calm and clear days. Point sampling for spectral data, cell counts, microscopy, and environmental sequencing was done on foot from the field station during calm and favorable weather conditions. Weather station data were collected for Photosynthetically Active Radiation (PAR), wind speed and temperature throughout the season and can be made available upon request.
Location	Sampling took place across the north-west part of Robert Island's ice cap at 50 - 130 m altitude above sea level. Robert Island is part of the South Shetland Islands to the north of the Antarctic Peninsula (-62.375248, -59.672096). All sampling was undertaken from the Luis Risopatron field station on Robert Island (INACH, Chile) (-62.378397, -59.700931) - https://www.inach.cl/expedicion-antartica/bases-chilenas-en-antartica-2/refugio-luis-risopatron/
Access & import/export	This research was funded under joint UKRI NERC grants NE/V000764/1 and NE/V000896/1 awarded to MPD, AG, CC, PC, PF, LP, AGS. Field access was organised and facilitated by the British Antarctic Survey (BAS) in co-ordination with the Chilean Antarctic Institute (INACH). All samples were exported from Antarctica under licence and imported into the United Kingdom under Articles 8 and 48(1) of Regulation (EU) 2016/2031 and Delegated Regulation (EU) 2019/829 and plant health authorisation no PH/2/2023.
Disturbance	Completion of an Environmental Impact Assessment (EIA) was carried out via BAS as it is a requirement of the Protocol on Environmental Protection to the Antarctic Treaty (1991) and the provisions of the Antarctic Act 1994, the Antarctic Act 2013 and accompanying Antarctic Regulations 1995/490. Strict biosecurity measures were implemented on arrival and on departure from Robert Island. All available steps were taken to minimise impacts on other terrestrial habitats (lichens, bryophytes, cyanobacteria) and associated megafauna (pinnipeds, birds) whilst undertaking fieldwork.

Reporting for specific materials, systems and methods

We require information from authors about some types of materials, experimental systems and methods used in many studies. Here, indicate whether each material, system or method listed is relevant to your study. If you are not sure if a list item applies to your research, read the appropriate section before selecting a response.

Materials & experimental systems

n/a	Involved in the study	
☒	☐	Antibodies
☒	☐	Eukaryotic cell lines
☒	☐	Palaeontology and archaeology
☒	☐	Animals and other organisms
☒	☐	Clinical data
☒	☐	Dual use research of concern
☒	☐	Plants

Methods

n/a	Involved in the study	
☒	☐	ChIP-seq
☒	☐	Flow cytometry
☒	☐	MRI-based neuroimaging

Plants

Seed stocks	*Report on the source of all seed stocks or other plant material used. If applicable, state the seed stock centre and catalogue number. If plant specimens were collected from the field, describe the collection location, date and sampling procedures.*
Novel plant genotypes	*Describe the methods by which all novel plant genotypes were produced. This includes those generated by transgenic approaches, gene editing, chemical/radiation-based mutagenesis and hybridization. For transgenic lines, describe the transformation method, the number of independent lines analyzed and the generation upon which experiments were performed. For gene-edited lines, describe the editor used, the endogenous sequence targeted for editing, the targeting guide RNA sequence (if applicable) and how the editor was*
